# Supplementary material for: Splice-Junction-Based Mapping of Alternative Isoforms in the Human Proteome
Source: Cell Rep. Author manuscript; Available in PMC 2020 Jan 15. (PMC6961840; doi:10.1016/j.celrep.2019.11.026)
Supplement: 3 [file NIHMS1546469-supplement-3.zip › DF2/PXD006675/LeftVentricle-98-O00468-TFVEYLNAVTESEK.pdf]

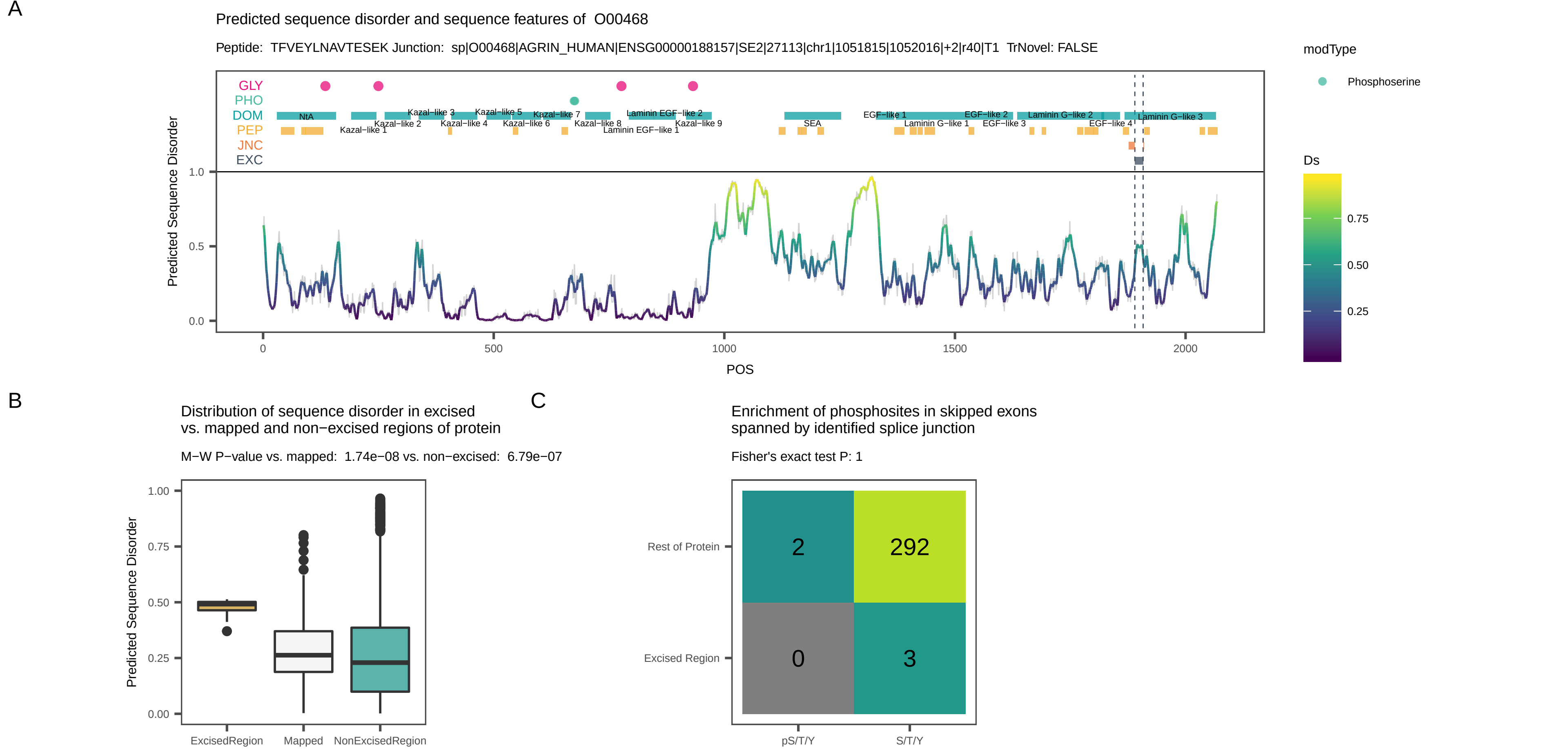

A
Predicted sequence disorder and sequence features of O00468
Peptide: TFVEYLNAVTESEK Junction: sp|O00468|AGRIN_HUMAN|ENSG00000188157|SE2|27113|chr1|1051815|1052016|+2|r40|T1 TrNovel: FALSE
GLY
PHO
DOM
PEP
JNC
EXC
NtA
Kazal−like 1
Kazal−like 2
Kazal−like 3
Kazal−like 4
Kazal−like 5
Kazal−like 6
Kazal−like 7
Kazal−like 8
Kazal−like 9
Laminin EGF−like 1
Laminin EGF−like 2
SEA
EGF−like 1
Laminin G−like 1
EGF−like 2
EGF−like 3
Laminin G−like 2
EGF−like 4
Laminin G−like 3
Predicted Sequence Disorder
POS
modType
Phosphoserine
Ds
B
Distribution of sequence disorder in excised vs. mapped and non−excised regions of protein
M−W P−value vs. mapped: 1.74e−08 vs. non−excised: 6.79e−07
Predicted Sequence Disorder
ExcisedRegion
Mapped
NonExcisedRegion
C
Enrichment of phosphosites in skipped exons spanned by identified splice junction
Fisher's exact test P: 1
Rest of Protein
Excised Region
2
292
0
3
pS/T/Y
S/T/Y
